# Rev-erb agonist improves adverse cardiac remodeling and survival in myocardial infarction through an anti-inflammatory mechanism

**DOI:** 10.1371/journal.pone.0189330

**Published:** 2017-12-12

**Authors:** Endin Nokik Stujanna, Nobuyuki Murakoshi, Kazuko Tajiri, DongZhu Xu, Taizo Kimura, Rujie Qin, Duo Feng, Saori Yonebayashi, Yukino Ogura, Fumi Yamagami, Akira Sato, Akihiko Nogami, Kazutaka Aonuma

**Affiliations:** Department of Cardiology, Faculty of Medicine, Graduate School of Comprehensive Human Sciences, University of Tsukuba, Tsukuba, Ibaraki, Japan; University of Cincinnati College of Medicine, UNITED STATES

## Abstract

Rev-erb α, known as nuclear receptor 1D1 (NR1D1), regulates circadian rhythm, modulates glucose and lipid metabolism, and inflammatory response. However, little is known about the effect of Rev-erb agonist on the progression of myocardial infarction (MI) and heart failure. To investigate it, wild-type male mice underwent sham-operation or permanent ligation of the left anterior descending coronary artery to create MI model. Rev-erb agonist SR9009 (100 mg/kg/day) or vehicle was intraperitoneally administered. Echocardiography was performed to evaluate cardiac function 1 week after surgery. The gene and protein expression levels in the left ventricles (LVs) were determined with real-time PCR, western blotting, and immunofluorescence. Moreover, immune cell infiltration into the LVs was analyzed by flow cytometry. Survival rate and reduced LV function were significantly improved by the treatment with SR9009 after MI. The expression level and plasma concentration of brain natriuretic peptide were significantly lower in MI mice treated with SR9009 (MI+SR) than in MI mice treated with vehicle (MI+V). Moreover, the mRNA expression levels of inflammatory-related molecules such as *Il6*, *Mcp1*, *Ly6g*, *Cd11b*, matrix metallopeptidase (*Mmp*)*9*, and the protein expression levels of phosphorylated NF-κB p65, phosphorylated ERK, and phosphorylated p38 were also significantly lower in MI+SR than in MI+V. Immunofluorescence intensity for MMP-9 was enhanced in the LVs, but was less so in MI+SR than in MI+V. Furthermore, infiltrations of neutrophils and proinflammatory macrophages in the LVs were dramatically increased in MI+V and were significantly suppressed in MI+SR. Rev-erb agonist SR9009 treatment inhibited post-MI mortality and improved cardiac function through modulating inflammation and remodeling process.

## Introduction

As a leading cause of death worldwide, myocardial infarction (MI) remains one of the most important clinical entities. After the onset of MI, cardiac tissue undergoes sequential molecular and cellular responses called remodeling [1]. During the acute phase, neutrophils and monocytes are recruited around the necrotic tissue, and they release inflammatory cytokines and matrix metalloproteinases (MMPs) [2]. Although these processes play important roles in the degradation of necrotic debris and subsequent scar formation, excess inflammatory response and MMP overproduction are likely to induce adverse cardiac remodeling, leading to cardiac dysfunction and rupture [3,4]. Despite the significant progress made on therapeutic strategies for MI in last few decades, mortality and morbidity remain high, and adverse cardiac remodeling after MI remains a critical issue to be solved. Therefore, continuous improvement in medications for the disease is still a major concern in global medical research.

Rev-erb belongs to a nuclear receptor superfamily containing two subgroups, Rev-erb α (NR1D1) and β (NR1D2), that regulate circadian rhythm and modulate glucose and lipid metabolism and inflammatory response [5,6]. Recently, SR9009 and SR9011 were developed as synthetic Rev-erb agonists and have been reported to improve hyperglycemia, dyslipidemia, and skeletal muscle oxidative capacity through modulation of mitochondrial number and oxidative function [6]. Moreover, long-term treatment with SR9009 was shown to reduce atherosclerotic plaque by decreasing the ratio of proinflammatory M1 macrophages to anti-inflammatory M2 macrophages in low-density lipoprotein (LDL) receptor-deficient mice fed a Western diet [7]. Thus, Rev-erb is expected to be a promising therapeutic target for metabolic syndrome and atherosclerotic disease. However, little is known about the pathophysiological roles of Rev-erb in the progression of MI and heart failure.

In this study, we aimed to clarify the roles of Rev-erb in post-MI remodeling and the effects of Rev-erb agonist SR9009 on cardiac function and survival after MI.

## Materials and methods

### Mouse model

After receiving approval from the Institutional Animal Experiment Committee of the University of Tsukuba, animal experiments were carried out humanely and in accordance with the Guide for the Care and Use of Laboratory Animals published by the US National Institutes of Health, the Regulation for Animal Experiments in our university, and the Fundamental Guideline for Proper Conduct of Animal Experiment and Related Activities in Academic Research Institutions under the jurisdiction of the Ministry of Education, Culture, Sports, Science and Technology of Japan.

We used 165 male wild-type C57BL6 mice purchased from CLEA Japan, Inc. (Tokyo, Japan) in total. At 12–14 weeks of age, mice were anesthetized with ketamine and xylazine, followed by tracheal intubation and artificial ventilation (MiniVent 845; Harvard Apparatus, Holliston, MA, USA). Next, a thoracotomy was performed to expose the heart, and MI was induced by the permanent ligation of the left anterior descending coronary artery (LAD) with 7–0 polypropylene suture passed about 1 mm from the inferior margin of the left atrial auricle. MI was confirmed by visual observation of myocardial color change and ST-segment elevation on the electrocardiogram recording. The sham-operated group was subjected to a similar procedure without ligation of the coronary artery. After closure of the thorax and recovery of spontaneous breathing, the mice were extubated and placed in a new box on the warm pad for recovery. Study period was within 2 weeks after surgery, and mice were monitored daily during the study. Among 165 mice used for all experiments, 58 mice died before sacrifice (40 mice died due to heart failure and/or arrhythmia including operational death, and 18 died due to cardiac rupture). If mice showed body weight loss of more than 20% and/or significant clinical signs due to the development of heart failure (e.g., increased respiratory rates, reduced activity, piloerection, hunched posture), they were immediately euthanized by intraperitoneal injection of 200 mg/kg sodium pentobarbital. Four mice were euthanized because of cardio-respiratory distress (weakness, fatigue, deep breathing, dyspnea). Thus, euthanized mice were considered as death due to heart failure.

SR9009 was purchased from Merck Millipore (Darmstadt, Germany) and was dissolved in 1% dimethyl sulfoxide (DMSO) in normal saline. We started intraperitoneal administration with SR9009 (100 mg/kg/day, the selection of the doses is based on previous study[7]) or vehicle (1% DMSO in normal saline) from 1 day before surgery. Consequently, mice were divided into four groups: sham-operated mice treated with vehicle (Sham+V), sham-operated mice treated with SR9009 (Sham+SR), MI mice treated with vehicle (MI+V), and MI mice treated with SR9009 (MI+SR). We first investigated mortality over the 14 days after MI and performed echocardiography at 7 days after MI and just before sacrifice. Next, we excised and collected the hearts of the mice sacrificed at 3 and 7 days after surgery for RNA and protein extraction and histochemical analysis, and those of the mice sacrificed at 1 and 5 days after surgery for flow cytometric analysis. For sacrifice, we used enough volume of sodium pentobarbital.

### Echocardiography

We obtained echocardiographic images from the parasternal long-axis view and short-axis view at the papillary muscle level under anesthesia with isoflurane using a Doppler echocardiographic system (Vevo 2100; Visual Sonics, Toronto, Canada). Accordingly, we determined left ventricular (LV) end-diastolic diameter (LVDd), LV end-systolic diameter (LVDs), fractional shortening (FS), LV ejection fraction (LVEF), interventricular septal thickness (IVST), posterior wall thickness (PWT) and LV mass. EF was calculated by the Teichholz method.

### Real-time PCR

RNA extraction and gene expression analysis were performed as previously described with minor modifications [8]. In brief, total RNA was extracted from the LV tissues using a RNeasy Fibrous Tissue Mini Kit (Qiagen, Venlo, Netherlands), and 2 μg of total RNA was reverse transcribed to cDNA with a High-Capacity cDNA Reverse Transcription Kit (Thermo Fisher Scientific, Inc., Waltham, MA, USA). The mRNA expression levels of the target genes were analyzed by an ABI Prism 7500 sequence detection system (Thermo Fisher Scientific). The commercially available gene-specific primers and probe sets were obtained from Integrated DNA Technologies (Coralville, IA, USA). The PCR mixture (10 μl total volume) consisted of primer and probe for each gene at 250 nM, and PrimeTime Gene Expression Master Mix (Integrated DNA Technologies). PCR amplification was performed in duplicate as follows: 1 cycle at 95°C for 10 min and 40 cycles at 94°C for 15 s and 60°C for 1 min. The primer and probe used in the study are mentioned as follows: *Nr1d1* (Mm.PT.58.17472803), *Nr1d2* (Mm.PT.58.31165809), *Nppb* (Mm.PT.58.8584045.g), *Il6* (Mm.PT.58.10005566), *Mcp1* (Mm.PT.58.42151692), *Ly6g* (Mm.PT.58.30498043), *Itgam* (*Cd11b*) (Mm.PT.5814195622), *Mmp9* (Mm.PT.58.10100097), *Col1a1* (Mm.PT.58.756.2513), *Col3a1* (Mm.PT.58.13848686). The quantitative values of target mRNA were normalized by 18S rRNA (4319413E, Thermo Fisher Scientific) expression. The results were obtained from 2–3 independent measurements (n = 4 in each group) performed in duplicate.

### Enzyme Immunoassay (EIA) for BNP

Plasma BNP concentration was measured by using a Brain Natriuretic Peptide EIA Kit (RAB0386-1KT, Sigma-Aldorich, Inc., St. Luise, MO, USA) according to the procedural manual. In brief, the samples and standards mixed with 10 pg/ml of biotinylated BNP peptide were added to the anti-BNP antibody-immobilized plate, where the biotinylated BNP peptide competed with unlabeled (endogenous) BNP for binding to the antibody. After incubating plate at 4°C overnight and washing wells, horseradish peroxidase (HRP)-streptavidin solution was added to each well, and incubated for 45 minutes at room temperature. After washing, tetramethylbenzidine (TMB) solution was then added to each well to visualize the immunoreaction. After adding stop solution, absorbance of each well was measured by using Varioskan LUX microplate reader (ThermoFisher) at 450nm. The result was obtained from one measurement (n = 4 in each group) performed in duplicate.

### Histology and immunofluorescence

The hearts were fixed with 4% paraformaldehyde, embedded in paraffin, sectioned into 4-μm-thick slices, and stained with Masson’s trichrome. Images were obtained by a digital microscope (Biozero BZ-X700; Keyence, Osaka, Japan), and collagen deposition was calculated by dividing the collagen depositing area to the total area. The analysis was performed using the ImageJ analysis software (Ver. 1.45, NIH, USA).

To evaluate the expression of MMP-9 on day 3, we performed immunofluorescence. After deparaffinization and antigen activation, sections were incubated with polyclonal rabbit anti-MMP9 antibodies (ab38898; Abcam, Cambridge, UK) at 4°C overnight. Next, sections were sufficiently rinsed in PBS and then incubated to Alexa Fluor 594-labeled goat anti-rabbit IgG (Thermo Fisher Scientific) at room temperature for 2 hours. After rinsing in PBS and mounting on slides with mounting medium with DAPI, the fluorescent images were captured by a digital fluorescent microscope (Biozero BZ-X700). The ratio of Alexa Fluora 594-positive area to total area was calculated with the ImageJ analysis software.

### Flow cytometry

Heart inflammatory cells were isolated and processed as described previously [9]. In brief, 1 and 5 days after MI or sham operation, hearts were excised, cut roughly, and dissociated by using gentleMACS Dissociator (Miltenyi Biotec, Bergisch Gladbach, Germany) in HBSS mixed with hyaluronidase, collagenase, and DNase I in accordance with the experimental protocol. Next, the cells were rinsed with PBS, dissolved with MACS buffer (Miltenyi Biotec), and passed through cell strainer (70μm) (Corning Inc., Corning, NY, USA). After removing red blood cells by ACK lysing buffer (Lonza Japan, Ltd., Tokyo, Japan), the cells were directly stained with fluorescence-conjugated antibodies as follows: FITC-conjugated anti-mouse Ly6g antibody, PE-conjugated anti-mouse CD11b antibody, and APC-conjugated anti-mouse CD45.2 antibody. To differentially investigate M1/M2 macrophages, the cells collected from mice 5 days after surgery were also stained with FITC-conjugated anti-mouse F4/80 antibody, PE-conjugated anti-mouse CD11b antibody, and APC-conjugated anti-mouse CD206 antibody. After 1 hour incubation at 37°, the cells were rinsed with PBS, diluted with MACS buffer, mixed with DAPI, and analyzed with a FACSCalibur instrument (Becton Dickinson Co., Franklin Lakes, NJ, USA), followed by analysis with FlowLogic software (Inivai Technologies, Mentone, Victoria, Australia). All antibodies were obtained from Bay Bioscience (Brookline MA, USA), Biolegend Inc., (San Diego, CA, USA), or Amersham Biosciences Corp., (Buckinghamshire, United Kingdom).

### Western blotting analysis

Western blotting was performed as described previously [8]. In brief, isolated LVs were homogenized in PRO-PREP protein extraction solution (iNtRON Biotechnology, Inc. Kyungki-Do, Korea), and the supernatants were used for western immunoblotting. Appropriate volumes of the samples (10 μg) were mixed with an equal volume of sample buffer, heated at 95°C for 5 min, and then subjected to SDS-PAGE using 4–15% gradient polyacrylamide gels (Bio-Rad Laboratory, Hercules, CA, USA). The proteins were transferred by semidry electroblotting from gels to polyvinylidene difluoride membranes. The blots were then blocked with the primary antibodies as follows: phospho-NF-κB p65 (Ser536 (#3033) and Ser468 (#3039)), NF-κB (L8F6) (#6956), phospho-p38 (Thr180/182) (#9211S), p38 mitogen activated protein kinase (MAPK) (#9212), phospho-p44/42 MAPK (Thr202/Thr204) (#9101S), p44/42 MAPK (#9102), β-actin (#4967) (all were purchased from Cell Signaling Technology, Inc., Danvers, MA, USA), and Rev-erb α (RS-14) (sc-100910, Santa Cruz Biotechnology, Inc, Dallas, TX, USA). The blots were incubated with an appropriate second antibody, horseradish peroxidase (HRP)-conjugated goat anti-rabbit IgG (ab6721, Abcam) or HRP-conjugated rabbit anti-mouse IgG (ab97046, Abcam). Immunoreactions were visualized with an enhanced chemiluminescence method (ECL Prime Western Blotting Detection; GE Healthcare, Southeast, UK). Densitometric analysis was performed on scanned immunoblot images with the ImageJ analysis software (Ver.1.45, NIH, USA). The ratios of densities of bands detected by using phosphorylated antibodies to those detected by using nonphosphorylated (total) antibodies or β-actin antibody was obtained from 2–3 independent measurements (n = 3 per group for each measurement).

### Statistical analysis

All data are expressed as mean ± SEM. Experimental groups were compared by one-way analysis of variance followed by Bonferroni’s test for multiple comparisons. When the results were not normally distributed, statistical analyses were performed using Kruskal-Wallis one-way analysis of variance. The effect of SR9009 on the survival of mice was analyzed by Kaplan-Meier methods and compared by log-rank test. Differences were considered statistically significant at p < 0.05. The analysis was performed using IBM SPSS version 21.0 software (IBM Co., Armonk, NY, USA).

## Results

### MI affects the expression of Rev-erb at mRNA and protein levels

**Fig 1:**
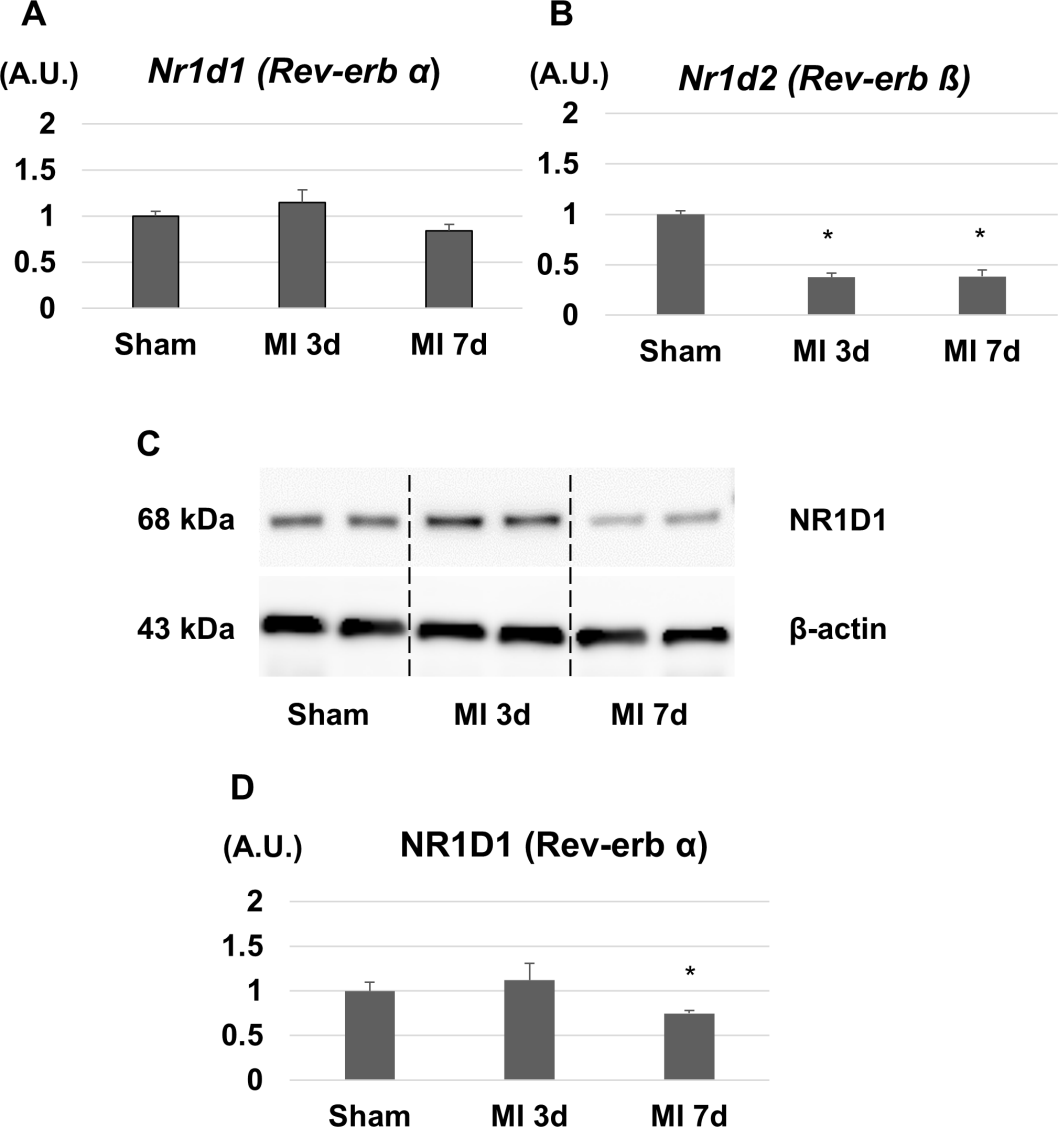
Gene and protein expression levels of Rev-erb in the left ventricular (LV) border area after myocardial infarction (MI). (A) The expression level of Rev-erb α mRNA in the LV was not significantly different between sham, 3 days, and 7 days after MI (n = 4 animals for each time point). (B) The expression level of Rev-erb β mRNA in the LV was significantly decreased 3 and 7 days after MI (n = 4 animals for each time point). (C) Western blotting analysis showed a significant decrease in the expression level of Rev-erb α 7 days after MI (n = 3 animals for each time point). The bar graphs show the group mean±SEM. *p<0.05 vs Sham.

First, we investigated the gene and protein expressions of Rev-erb in the LVs after MI. The mRNA expression level of Rev-erb α in the LVs was slightly changed but not significantly different between sham, day 3, and day 7 after MI (Fig 1A). The protein expression level of Rev-erb α was slightly increased on day 3 whereas it was significantly decreased on day 7 after MI (Fig 1C). On the other hands, the mRNA expression level of Rev-erb β was significantly decreased on day 3 and 7 after MI (Fig 1B).

### SR9009 suppresses post-MI mortality and improves cardiac function

**Fig 2:**
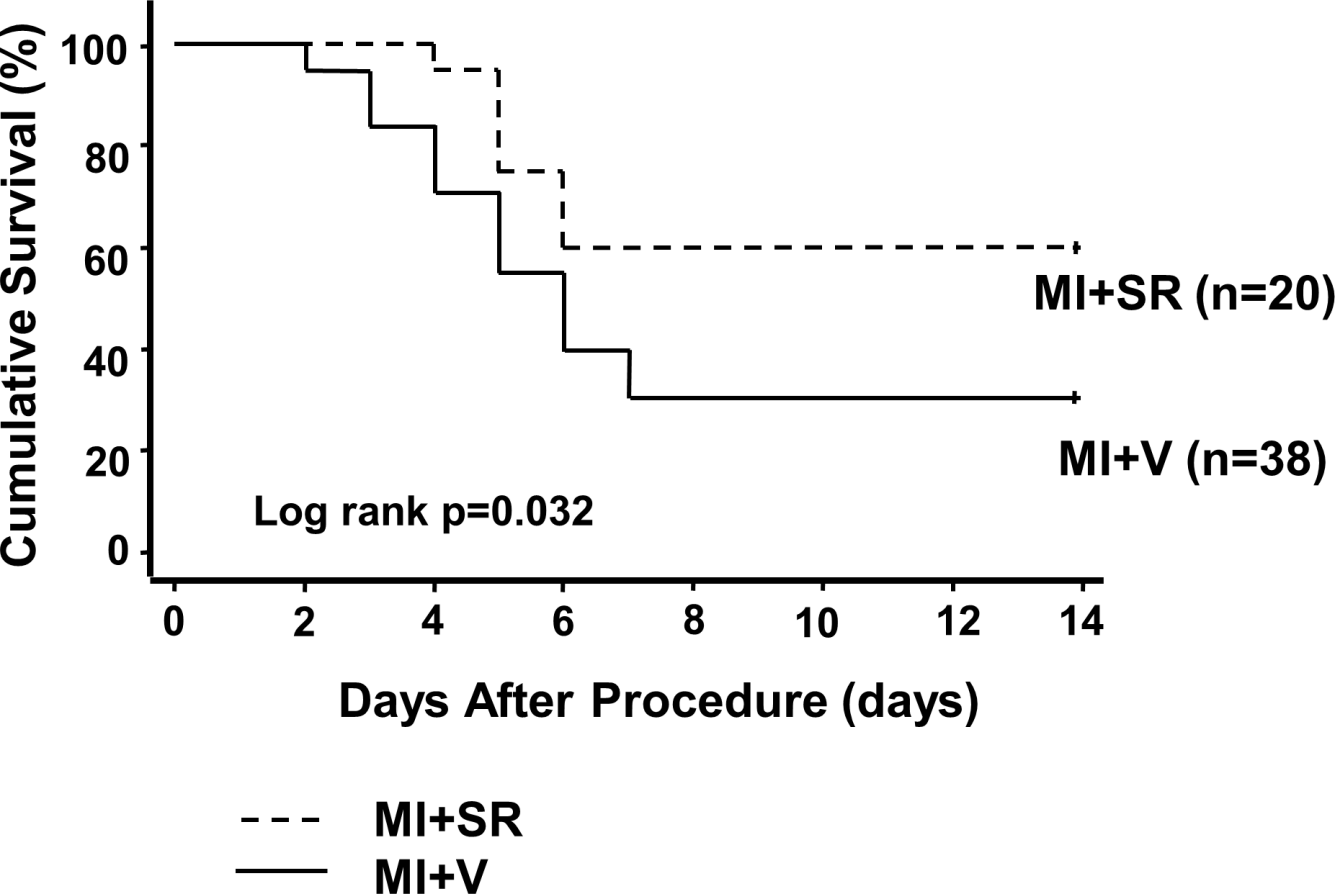
Kaplan-Meier survival curves after myocardial infarction. Survival rate was significantly higher in MI+SR than in MI+V (60.0% vs 31.6% at day 14 after myocardial infarction; log-rank test, p = 0.032 compared between MI+SR and MI+V). Solid line denotes the survival curve of MI+V and dashed line denotes of MI+SR.

We next investigated survival after MI with or without SR9009 treatment. After permanent ligation of the LAD in 58 mice (38 in MI+V; 20 in MI+SR), 18 mice (15 in MI+V; 3 in MI+SR) died because of ventricular rupture that occurred between 3 and 7 days after MI. Meanwhile, 16 mice (11 in MI+V; 5 in MI+SR) died without bleeding in the thoracic cavity. We assumed they died either due to acute heart failure or cardiac arrhythmia. The survival rate of MI+V group was 31.6%, while it was significantly higher in MI+SR (60.0%, log-rank p<0.05; Fig 2).

**Fig 3:**
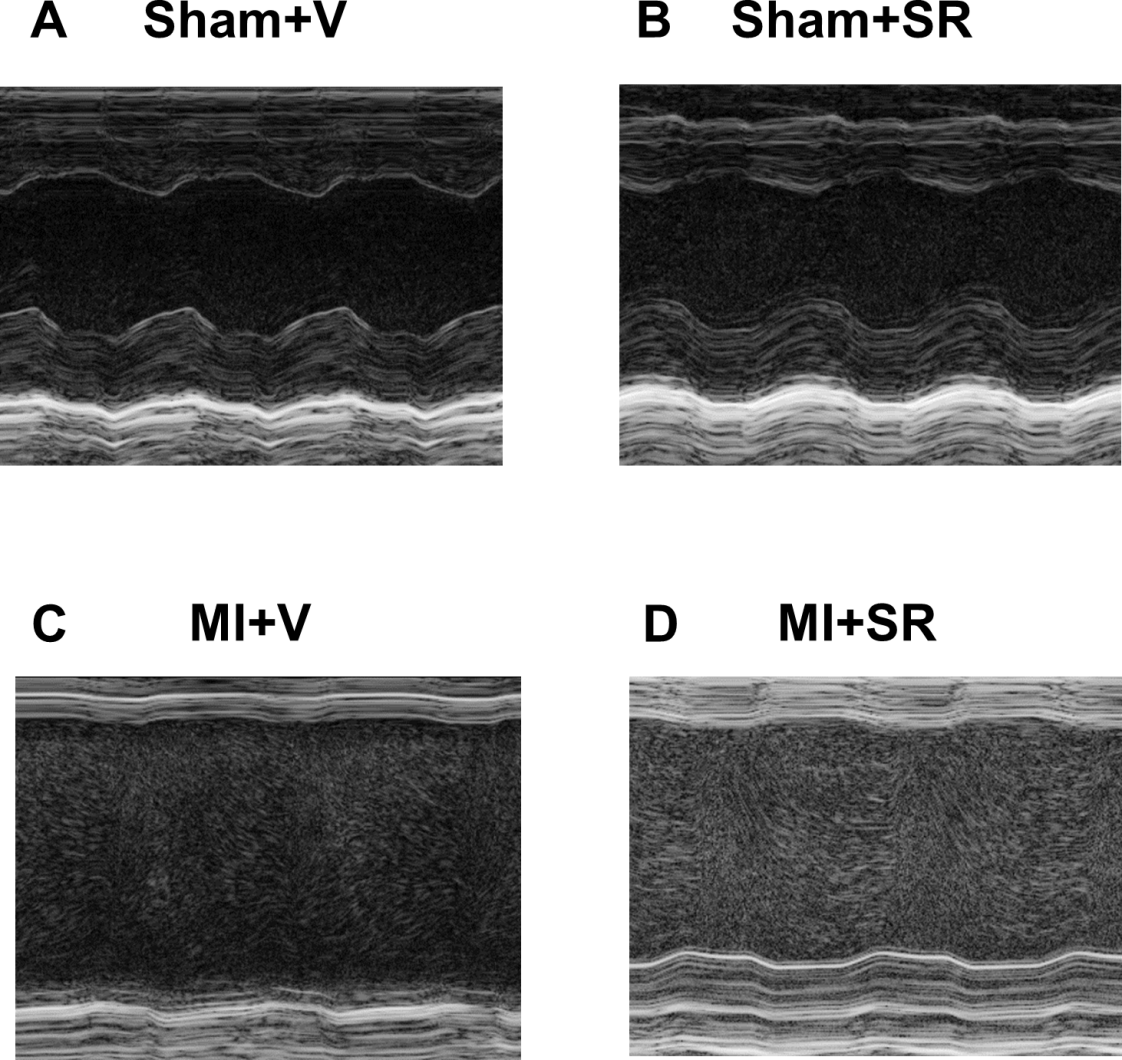
M-mode echocardiographic analysis. The representative M-mode findings of the left ventricular short-axis view on day7 after MI: (A) Sham+V, (B) Sham+SR, (C) MI+V, and (D) MI+SR.

To assess cardiac function after MI, we performed echocardiographic analysis 7 days after surgery in each group (Fig 3A–3D). Echocardiographic analysis revealed that SR9009 treatment significantly improved LVEF and FS (Table 1.). LVDd, Ds, and LV mass were slightly lower in MI+SR, but these were not significantly different between MI+V and MI+SR (Table 1.). The ratios of heart weight to BW, LV weight to BW, and lung weight to BW were not significantly different between MI+V and MI+SR (Table 1.).

**Table 1:** Tissue weights and echocardiographic parameters.

	Sham+V	Sham+SR	MI+V	MI+SR
	n = 4	n = 4	n = 9	n = 7
BW	24.02±0.62	25.35±0.23	23.77±0.75	23.39±0.83
Heart	125.8±2.45	139.45±6.55	157.38±9.42*	148.97±13.46
HW/BW	5.24±2.45	5.49±0.24	6.78±0.46*	6.30±0.40
LV/BW	4.29±0.05	4.44±0.15	5.42±0.36*	5.35±0.51*
Lung/BW	5.38±0.21	5.19±0.13	8.25±1.08*	7.20±0.87*
LVDd (mm)	3.16±0.19	2.78±0.24	5.25±0.21*	4.88±0.26*
LVDs (mm)	2.13±0.23	1.87±0.30	4.97±0.24*	4.36±0.30*
FS (%)	31.67±4.74	32.29±4.10	6.23±0.92*	11.07±2.04*†
EF (%)	64.67±7.66	61.71±6.27	13.85±2.02*	23.78±4.21*†
IVST (mm)	0.725±0.02	0.69±0.11	0.67±0.06	0.74±0.03
PWT (mm)	0.62±0.10	0.90±0.07	0.68±0.04	0.60±0.11
LV Mass (mg)	83.12±12.11	85.72±11.72	156.02±21.33*	147.30±11.64*

LV ejection fraction (EF) and fractional shortening (FS) were significantly higher in MI+SR than in MI+V, whereas LV end-diastolic diameter (LVDd), LV end-systolic diameter (LVDs) and LV mass were not significantly different between MI+V and MI+SR, and the ratios of heart weight to BW, left ventricular (LV) weight to BW, and lung weight to BW were also not significantly different between MI+V and MI+SR (n = 4 in each sham group, n = 9 animals in MI+V group, and n = 7 animals in MI+SR group). The bar graphs show the group mean±SEM.

*p<0.05 vs Sham+V

†p<0.05 vs MI+V

### SR9009 does not affect collagen deposition after MI

**Fig 4:**
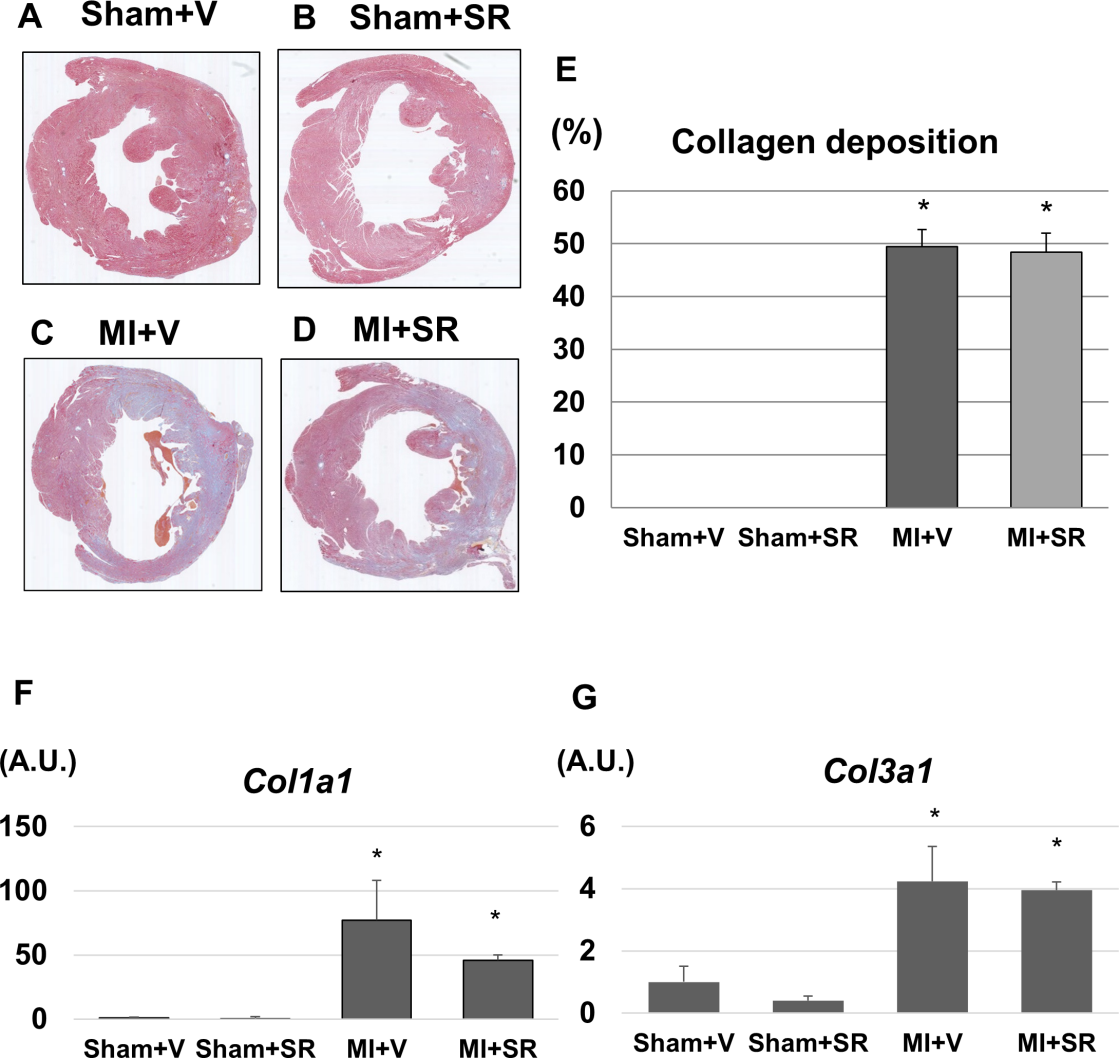
Representative photographs of the left ventricles at papillary muscle level. (A) Sham+V, (B) Sham+SR, (C) MI+V, and (D) MI+SR. (E) shows the ratio of collagen depositing area to total area. There was no significant difference in collagen deposition between MI+V and MI+SR (n = 4 animals in each group). (F) The mRNA expression level of collagen type Ia1 (*Col1a*) was significantly increased in MI+V, and was tend to be decreased in MI+SR, but not significantly different between MI+V and MI+SR. (G) The mRNA expression level of collagen type IIIa1 (*Col3a*) was also significantly increased in MI+V, and was not significantly different between MI+V and MI+SR (n = 4 animals in each group).

Representative photomicrographs of LV tissues are shown in Fig 4A–4D. LV size and wall thickness were similar between Sham+V and Sham+SR. Although collagen deposition was observed in large areas of the LV of both MI+V and MI+SR, there were no obvious histological differences between the two groups. Quantitative analysis revealed that there was no significant difference in collagen depositing area between MI+V and MI+SR (Fig 4E). LV gene expression levels of *Col1a* and *Col3a* were markedly upregulated 7 days after MI. SR9009 treatment reduced the *Col1a* expression, but there were no significant differences in mRNA expression levels of *Col1a* and *Col3a* between MI+V and MI+SR (Fig 4F and 4G).

### SR9009 inhibits the expressions of natriuretic peptide, inflammatory cytokines, and MMP-9

**Fig 5:**
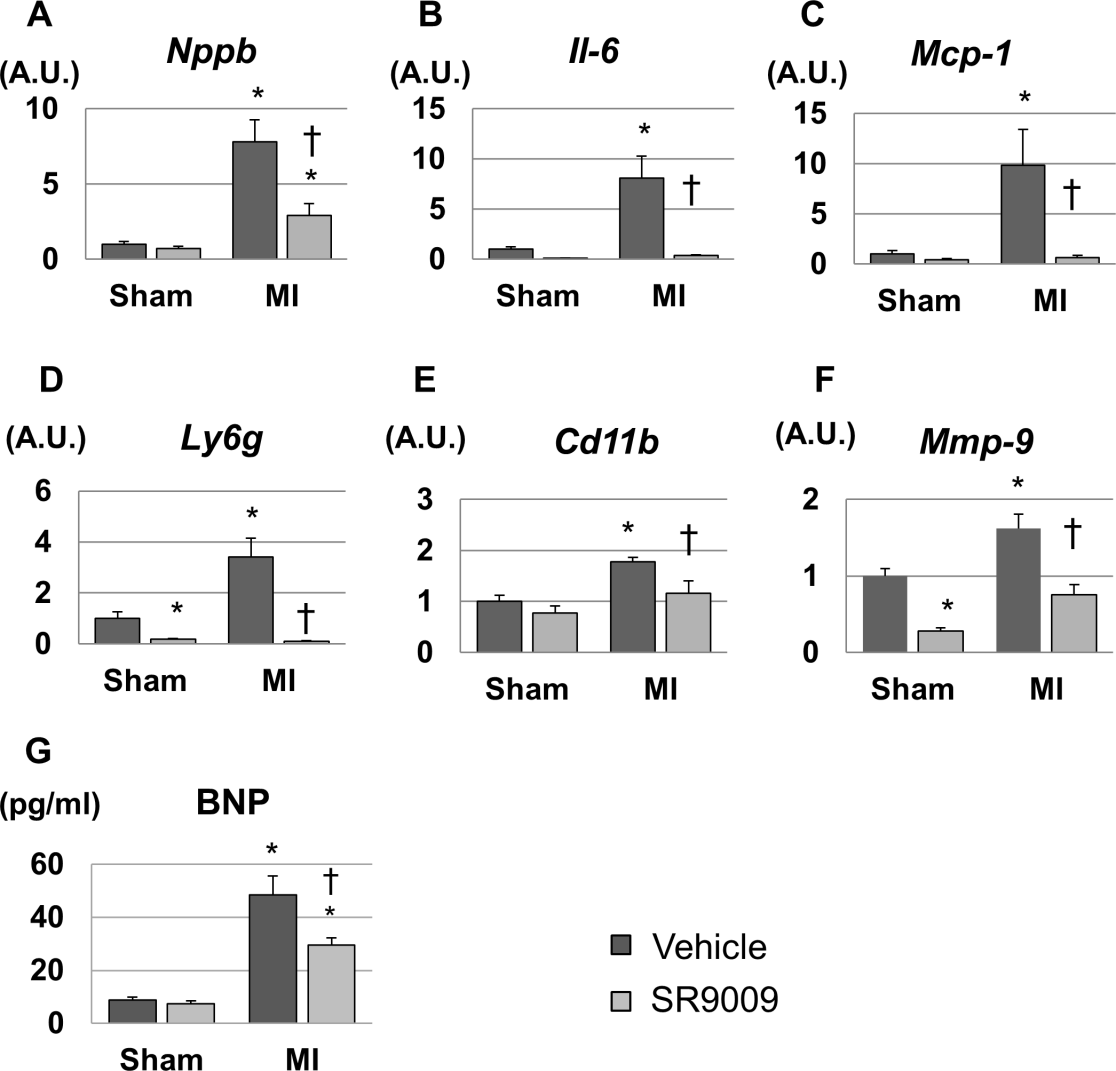
Gene expression levels of the left ventricles analyzed by real-time PCR and plasma BNP concentration. (A) The mRNA expression level of natriuretic peptide precursor B (*Nppb*) was significantly higher in MI+V than in sham, and was significantly lower in MI+SR than in MI+V (n = 4 animals in each group). The expression levels of (B) *Il6*, (C) *Mcp1*, (D) *Ly6g*, and (E) *Cd11b* were significantly increased in MI+V and were markedly decreased in MI+SR. (F) Matrix metallopeptidase (*Mmp*)*9* was also significantly lower in MI-SR than in MI-V. (G) Plasma BNP concentration was also significantly higher in MI than in sham, and was significantly lower in MI+SR than in MI+V. The bar graphs show the group mean±SEM. *p<0.05 vs Sham+V; †p<0.05 vs MI+V.

We investigated mRNA expression levels in the LV by using real-time PCR. The mRNA expression level of natriuretic peptide precursor B, which encodes a marker of heart failure brain natriuretic peptide (*Bnp*), was 8-fold higher in MI+V compared to Sham+V, and it was significantly lower in MI+SR than in MI+V (Fig 5A). Moreover, the left ventricular expression levels of inflammatory cytokines *Il6* and *Mcp1*, neutrophil-related surface antigen *Ly6g*, and monocyte/macrophage-related surface antigen *Cd11b* were increased after MI, and these levels were dramatically decreased by SR9009 treatment (Fig 5B–5E). The mRNA expression level of *Mmp9*, a major extracellular matrix protein playing a critical role in post-MI remodeling, was also increased in MI+V and was significantly decreased in MI+SR compared with MI+V (Fig 5F). Moreover, plasma BNP concentration was also significantly higher in MI+V than in Sham+V, and it was significantly lower in MI+SR than in MI+V (Fig 5G). These results suggested that elevated BNP, infiltration of neutrophils and monocytes/macrophages, and productions of inflammatory cytokines and extracellular matrix were reduced by the treatment with SR9009 during the acute phase of MI.

**Fig 6:**
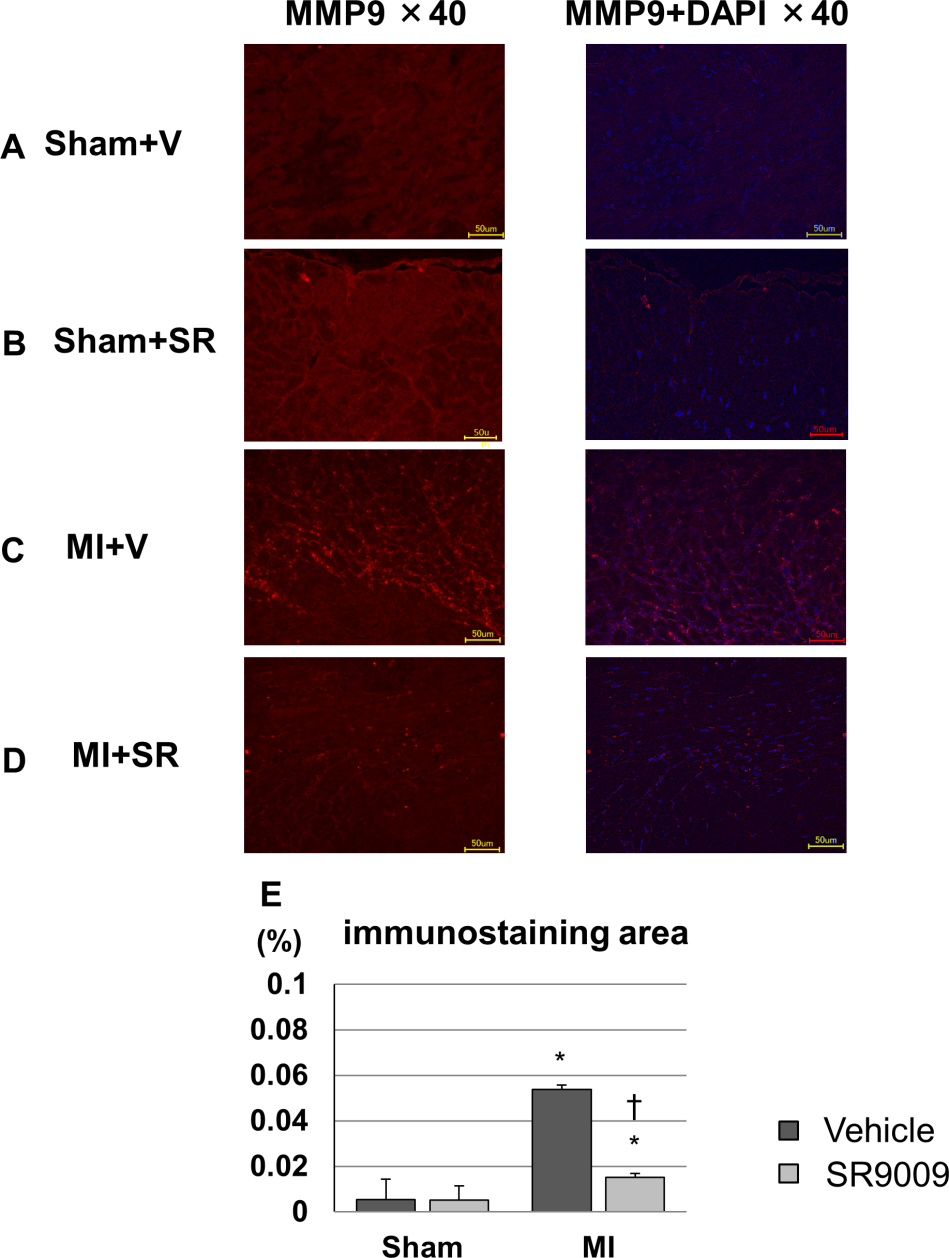
Representative immunofluorescence images of the left ventricles stained with MMP-9. (A) Sham+V, (B) Sham+SR, (C) MI+V, and (D) MI+SR. MMP-9 was stained with Alexa Fluor 594 (red) and nuclei were stained with DAPI (blue). All images show 40× magnification. Sham groups showed low immunofluorescence intensity, and MMP-9 was strongly detected in the infarct and border areas in the MI groups. MI+SR showed less intensity compared with MI+V. (E) The ratio of immunofluorescence-positive area to total area was significantly increased in MI+V, and was markedly decreased in MI+SR (n = 4 animals in each group). The bar graphs show the group mean±SEM. *p<0.05 vs Sham+V; †p<0.05 vs MI+V.

To confirm the protein expression level of MMP-9, we performed immunofluorescence. Immunofluorescence intensity for MMP-9 could be weakly detected in both of the sham groups, and it was enhanced mainly in the interstitial tissue of the border area after MI. MI+SR displayed less intensity of MMP-9 compared to that in MI+V (Fig 6). Quantitative analysis revealed that significant increase in immunofluorescence-positive area in MI+V was reduced by SR9009 treatment.

### SR9009 reduces infiltration of neutrophils and M1 macrophages into the infarcted myocardium

**Fig 7:**
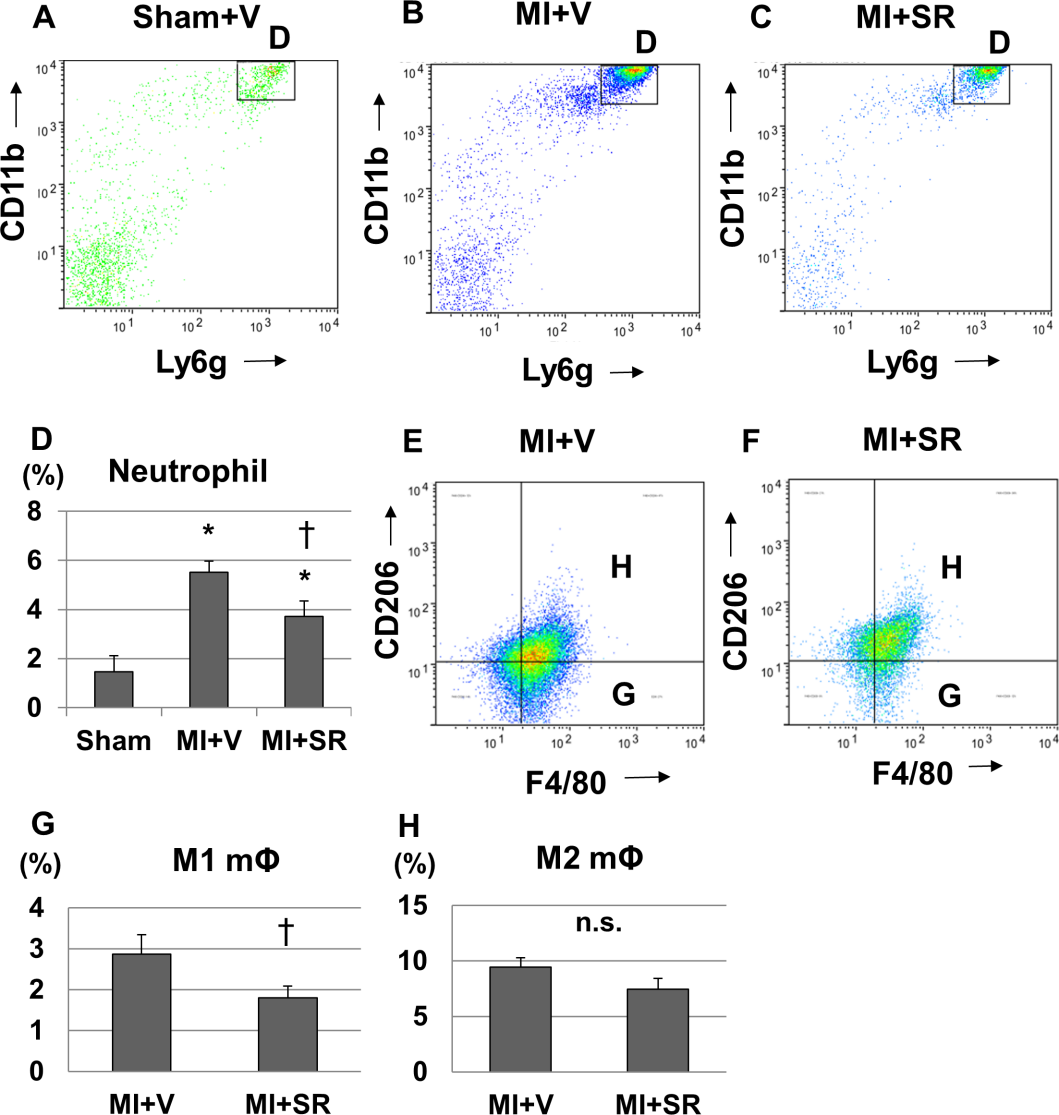
Flow cytometric analysis. Flow cytometric analysis of the infiltration of inflammatory cells into the infarct and border areas at 1 day after myocardial infarction (MI) (n = 5 in each group). The representative plots (gated on CD45+ and expanded into Ly6G and CD11b) of (A) Sham+V, (B) MI+V, and (C) MI+SR9009 are shown. (D) The percentage of CD45+CD11b+Ly6G+ cells (neutrophils) to CD45+ cells was significantly lower in MI+SR than in MI+V, suggesting that neutrophil infiltration was reduced by SR9009 treatment. Flow cytometric analysis of M1/M2 macrophage infiltration into the infarct and border areas at 5 days after MI (n = 5 in each group). The representative plots (gated on CD11b and expanded into F4/80 and CD206) of (E) MI+V, and (F) MI+SR9009 are shown. (G) The percentage of F4/80+CD206- cells to live cells was significantly lower in MI+SR than in MI+V although the percentage of F4/80+CD206+ cells to live cells was not significantly different between MI+V and MI+SR, suggesting that proinflammatory M1 macrophage infiltration was reduced by SR9009 treatment. The bar graphs show the group mean±SEM. *p<0.05 vs Sham+V; †p<0.05 vs MI+V.

To further investigate the effect of SR9009 on the infiltration of inflammatory cells into the LV during the acute phase of MI, we performed flow cytometric analysis. CD45+CD11b+Ly6G+ cells (neutrophils) were dramatically increased in the heart 1 day after MI from MI+V compared to Sham+V (Fig 7A and 7B). SR9009 treatment decreased the infiltrations of CD45+CD11b+Ly6G+ cells 1 day after MI (Fig 7C). Quantitative analysis revealed a significant decrease in the percentage of CD45+CD11b+Ly6G+ cells to CD45+ cells (Fig 7D). These results suggested that neutrophil influx was reduced by the treatment with SR9009 during the acute phase.

We next investigated the infiltrations of M1/M2 macrophages in the heart 5 days after MI. The percentage of CD11b+F4/80+CD206+ cells (M2 macrophages) to live cells was not significantly different between MI+V and MI+SR 5 days after MI; however, the percentage of CD11b+F4/80+CD206- cells (M1 macrophages) to live cells was significantly lower in MI+SR than in MI+V (Fig 7E–7H).

### SR9009 modulates NF-κB and MAPKs signalling pathway in the LV

**Fig 8:**
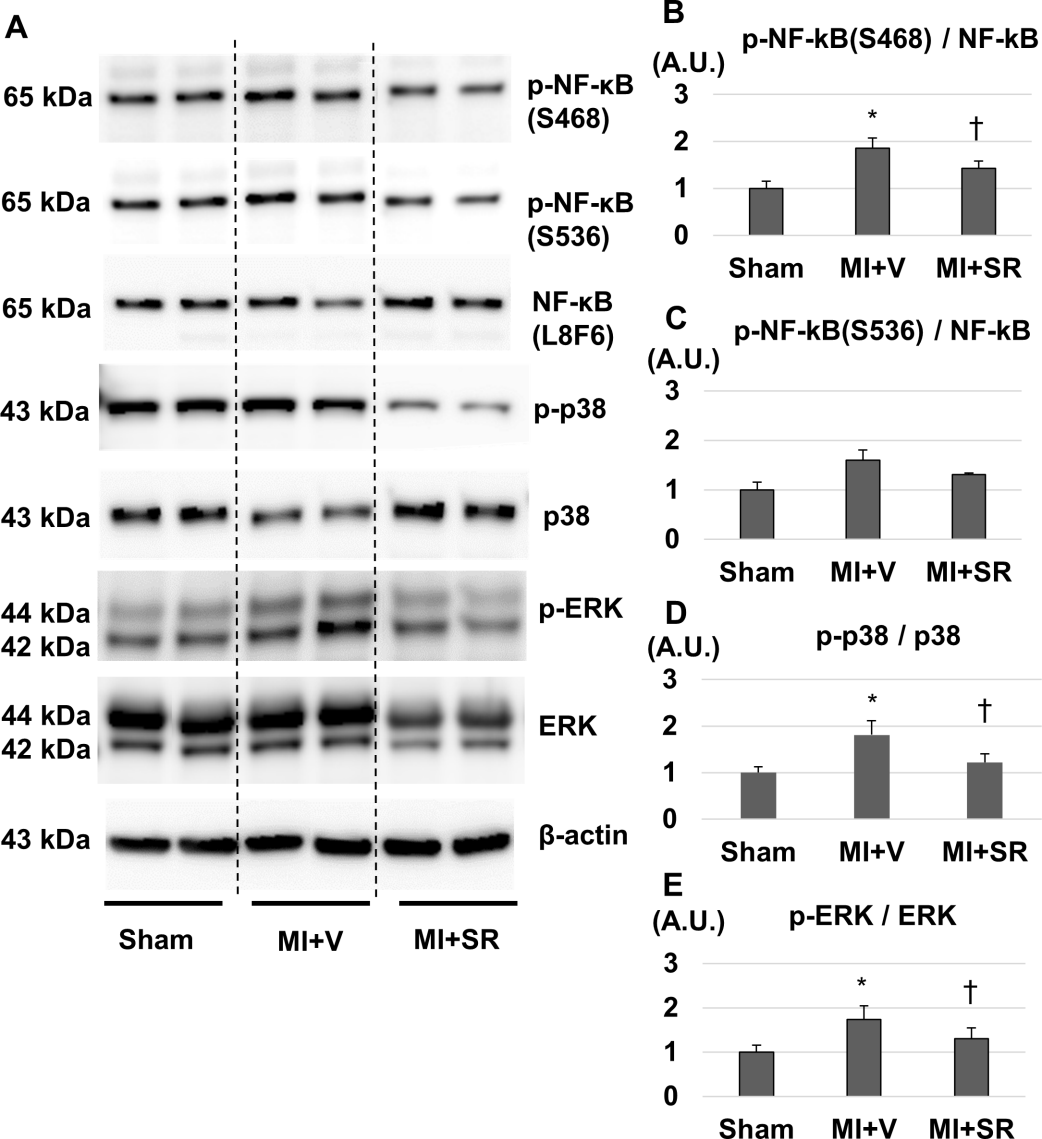
Western blotting analysis. (A) Representative images of western blotting for phospho-NF-κB p65 (S468 and S536), NF-κB, phospho-p38, p38, phospho-ERK, ERK, and β-actin. (B) The ratio of phospho-NF-κB (S468) to NF-κB was significantly lower in MI+SR than in MI+V. (C) The ratio of phospho-NF-κB (S536) to NF-κB had a similar tendency, but no significant difference. (D) The ratio of phospho-p38 to p38 was significantly lower in MI+SR than in MI+V. (E) The ratio of phospho-ERK to ERK was also significantly lower in MI+SR than in MI+V. (n = 3 animals in each group). The bar graphs show the group mean±SEM. *p<0.05 vs Sham+V; †p<0.05 vs MI+V.

Rev-erb α has been reported to repress cytokine productions not only directly through a Rev-erb α-binding motif but also indirectly through NFκB- and/or MAPK-signaling pathway [10,11]. Therefore, we performed western blotting to evaluate whether SR9009 treatment influenced NF-κB and MAPKs in the LV after MI. SR9009 treatment suppressed the expression of serine 468-phosphorylated (S468) and serine 536-phosphorylated (S536) NF-κB p65 (Fig 8A). Quantitative analysis revealed that the ratio of phospho-NF-κB p65 (S468) to NF-κB p65 was significantly lower in MI+SR than in MI+V (Fig 8B). SR9009 treatment also suppressed phosphorylations of p38 and p44/42 (ERK) (Fig 8A). The ratios of phosphorylated p38 to p38 and phosphorylated ERK to ERK were significantly lower in MI+SR than in MI+V (Fig 8D).

## Discussion

In this study, we showed for the first time, to our knowledge, that Rev-erb agonist SR9009 improved LV function and survival after MI. Moreover, we also showed that Rev-erb agonist decreased *Il6* and *Mcp1* production, *Mmp9* expression, NF-κB and MAPKs activations, and neutrophil/M1 macrophage infiltrations in infarct and border myocardium during the acute phase of MI. These results suggested that the pharmacological activation of Rev-erb exerts beneficial effects on the acute process of post-MI remodeling by strongly inhibiting cytokine production and inflammatory cell infiltration into the infarcted heart.

Inflammatory response after MI is a double-edged sword, and how to regulate inflammation to prevent adverse cardiac remodeling is an important issue to be considered when developing a new treatment for heart failure [12–14]. In the present study, we showed that the expression levels of inflammatory cytokines including *Mcp1* and *Il6* were dramatically suppressed by SR9009 treatment in the LV during the acute phase of MI, and that of *Mmp9* was also suppressed by SR9009 treatment. Moreover, we also showed that NF-κB and MAPKs activation were inhibited by the treatment with SR9009. These findings are consistent with the previous studies [10,11,15,16]. Sato et al. reported that activated Rev-erb α repressed *Il6* expression not only directly through a Rev-erb α binding motif but also indirectly through suprression of NF-κB activity [10]. They also reported that Rev-erb α regulated the inflammatory function of macrophages through suppression of MCP-1-acitivating ERK and p38 signalling pathway [11]. *Il6* receptor antagonist has been reported to reduce leukocyte and macrophage infiltration and attenuate MMP activation, leading to a marked improvement in LV remodeling and survival after MI [17]. Moreover, targeted deletion of *Mcp1* attenuated LV remodeling after MI through decreases in macrophage infiltration and MMP activation [18,19]. MMP-9 is known as a major subtype of metalloproteinase and plays a crucial role in post-MI remodeling. In the acute inflammatory phase after MI, neutrophils and monocytes are considered to be the predominant source of MMPs. Higher production of MMPs is more likely to cause LV wall thinning and dilatation, consequently leading to heart failure and cardiac rupture. It has been reported that genetic and pharmacological deletion of MMP-9 prevents adverse LV remodeling after MI [20,21]. Moreover, previous study reported that Rev-erbs repressed MMP-9 expression at a distance by regulating enhancer-directed transcription [16]. Thus, the present study, taken together with the findings of previous reports, suggests that the strong inhibitory effect of SR9009 on the production of inflammatory cytokines such as *Il6* and *Mcp1* attenuates neutrophil and M1 macrophage infiltrations and MMP-9 production, at least partially through suppression of NF-κB, ERK, or p38 signalling pathway, leading to inhibition of the vicious circle of proinflammatory amplification, the development of adverse LV remodeling, and cardiac rupture.

MAPKs are known as important signaling pathway that regulates cell survival, apoptosis, and proliferation. In the present study, phosphorylated ERK and phosphorylated p38 were significantly decreased by SR9009 treatment in the LV after MI. ERK activation is thought to inhibit apoptotic loss of cardiomyocytes and promote cardiac fibrosis. On the other hands, p38 activation has been implicated in the progression of apoptosis and cardiac dysfunction, leading to pathological remodeling although still controversial [22].

Despite a significant improvement of LV function by SR9009 treatment, there was no significant difference in collagen deposition between MI+V and MI+SR. The size of collagen deposition area is mainly determined by the size of the necrotic area, the wound healing response, and chronic remodeling [23]. Among them, the size of the necrotic area is the most critical for determination of the scar size in acute phase of MI, especially without reperfusion. Because we employed mice model with the permanent ligation of the proximal LAD in this study, necrotic area was relatively extensive and generally constant in each mouse. Therefore, the area of collagen deposition was mostly influenced by the size of necrotic area with or without treatment. However, because histological data were only obtained from the samples collected within 1 week after MI, further studies are needed to evaluate the effect of Rev-erb agonist in collagen deposition in chronic phase of MI.

Rev-erb α regulates not only inflammation but also mitochondrial content and oxidative capacity. It has been reported that expression levels of phosphorylated AMPK, sirtuin 1, PPAR-gamma coactivator (PGC)-1α, and mitochondrial transcription factor A (TFAM) were significantly lower in the skeletal muscle from homogenous Nr1d1-knockout (*Nr1d1*^*-/-*^) mice compared to wild-type mice [6]. Consequently, mitochondrial DNA content was significantly lower in skeletal muscle from *Nr1d1-/-* mice, and VO_2_ max during exercise was significantly lower in *Nr1d1*^*-/-*^ than in wild-type mice. By contrast, pharmacological activation or skeletal muscle-specific Rev-erb α overexpression resulted in the opposite phenotypes. These findings suggested that Rev-erb α controls mitochondrial biogenesis and respiration in the skeletal muscle through the *Ampk-Sirt1–Pgc1α-Tfam* signaling pathway. Several studies reported that activation of AMPK, such as treatment with metformin, protects the heart against cardiac stress such as ischemia by regulating glucose and fatty acid metabolism [24,25]. Therefore, favorable effects of Rev-erb agonist SR9009 on cardiac function may be partially attributed to activation of this pathway and modulation of the metabolic process[26–30]. Further studies are needed to clarify the effect of the Rev-erb agonist on cardiac metabolism after MI, especially during the chronic phase.

SR9009 and SR9011 were synthesized as novel Rev-erb agonists, and they have been reported to improve hyperglycemia, dyslipidemia, and skeletal muscle oxidative capacity by modulating mitochondrial number and oxidative function in mice [6]. Moreover, long-term treatment with SR9009 could reduce atherosclerotic plaque by decreasing the ratio of pro-inflammatory M1 macrophages to anti-inflammatory M2 macrophages in LDL receptor-deficient mice fed a Western diet [7]. In terms of clinical implications, the present study, taken together with the previous reports, suggested that Rev-erb agonists can be expected to be promising novel drugs not only for metabolic syndrome and atherosclerotic disease but also for MI and heart failure. For example, the drugs may be useful for secondary prevention in patients with MI complicated with metabolic syndrome.

The present study included the following limitations. First, we only studied the acute phase of MI using a mouse model with permanent ligation of the coronary artery. Further studies are needed to elucidate the long-term effects of Rev-erb agonist and/or in an ischemia/reperfusion model. Second, although we administered only one dose of SR9009 (100 mg/kg/day) according to the previous study [5], we may need another dose to investigate whether dose dependency exists. Moreover, we tried to start the treatment with SR9009 on the next day after MI surgery; however, the survival rate and LV function did not reach significant improvement. Longer periods of severe ischemia (usually more than 6-hour ischemia due to complete coronary occlusion) is considered to cause loss of most cardiomyocytes in the subendocardial region of the ischemic area [31]. “Late-start treatment” with SR9009 may not exert enough inhibitory effects on cardiac cell death although a single injection of 100mg/kg of SR9009 quickly alters circadian pattern and metabolic gene expressions in mice [5]. As clinical implications, Rev-erb agonists may be useful for secondary prevention in patients with prior MI or metabolic syndrome with high risk for coronary artery disease although late-start treatment with Rev-erb agonist after the onset of MI may not be enough to improve clinical prognosis. Third, this study suggested that the favorable effect of SR9009 on post-MI remodeling was mainly attributed to the strong attenuations of inflammatory cytokine production and MMP-9 production. However, another mechanism such as alteration of metabolic processes and/or circadian rhythm may contribute to the results. Further studies are desirable to investigate the precise mechanism of the effect of Rev-erb activation on post-MI remodeling using antagonist or gene-manipulated mice models.

In conclusion, Rev-erb agonist SR9009 could improve cardiac function and survival in a mouse model of acute MI by inhibiting cytokine production and inflammatory cell infiltration. Rev-erb α is expected to be a promising therapeutic target not only for metabolic syndrome and atherosclerotic disease but also for MI and heart failure.
